# Health Promotion Behavior among Older Korean Family Caregivers of People with Dementia

**DOI:** 10.3390/ijerph18084123

**Published:** 2021-04-13

**Authors:** Aram Cho, Chiyoung Cha

**Affiliations:** 1 College of Nursing Science, Ewha Womans University, Seoul 03760, Korea; pinkalice91@naver.com; 2 College of Nursing Science & Ewha Research Institute of Nursing Science, System Health & Engineering Graduate School, Ewah Womans University, Seoul 03760, Korea

**Keywords:** dementia caregiver, health promotion behavior

## Abstract

People adopt health promotion behaviors to promote their health as they interact within the environment. The purpose of this study was to examine factors influencing health promotion behaviors among older adults caring for family members with dementia. For this cross-sectional study, data from 135 older adults who were the main caregivers were collected at an outpatient clinic at a university hospital in the capital city of South Korea between September and October in 2020. Sociodemographic characteristics, caregiver-related characteristics, dementia knowledge, fear of dementia, and health promotion behaviors were measured. Univariate analysis revealed that the level of health promotion behaviors differed by age, sex, educational level, monthly income, relationship with the family member with dementia, and cohabitation with family members with dementia. In the multivariate analysis, a hierarchical multiple regression model explained 33.9% of the variance. Sex, duration of caregiving, use of long-term care service, and fear of dementia predicted health promotion behavior. A strategic tailored care plan for target population is needed to improve the health promotion behavior of older adults caring for family members with dementia.

## 1. Introduction

With an aging global society, dementia is one of the fastest growing diseases in the world. The World Alzheimer report estimates that the global population with dementia will increase by threefold in thirty years [1]. People living with dementia experience increasing difficulties with activities of daily living (ADL) over time [2] and need assistance from their family members. Family caregivers for people with dementia are often imposed with physical, psychological, and economical burdens [3]. The issues of family caregivers are expected to increase with the growing population of people with dementia. Individuals who care for people with dementia are known to experience various health issues such as headache, indigestion, neuralgia, fatigue, sleep deprivation, anxiety, and depression [4]. Considering the magnitude of the problem with family caregivers, the World Health Organization (WHO) included the support for health of caregivers of people with dementia in their action plan [5]. By supporting health promotion, which involves activities that influence public health, empowerment of the community could be achieved [6].

However, it is hard for family caregivers to attend to their health because most of the time they have limited time and energy to focus on their own health [6]. Deterioration of the health of a family caregiver increases the caregiving burden [7], which in turn might negatively influence the people with dementia. Moreover, in Asian countries, family members often take the full responsibility of caring for other family members [8], which has drawn the attention of health professionals and policy makers.

In an aging society, family caregivers of people with dementia are also aging. Previous studies have reported that the average age of family caregivers of people with dementia is between 65 and 66 years [7,9,10,11]. The issues with aging caregivers are that they might also have their own health issues to attend to, might have limited information on dementia, and they might develop a fear of dementia. In addition, family caregivers of people with dementia are known to have decreased health-related quality of life [12], which increases the importance of health promotion. Moreover, older family caregivers’ lack of knowledge about and fear of dementia might also contribute to their health promotion behaviors.

Family caregivers with knowledge about dementia is important because without proper information, it is hard to cope with unexpected issues with caregiving [13]. Although we live in a society where information can be accessed easily, studies of family members of people with advanced dementia have revealed that they only have a lay understanding of dementia and do not know the trajectory of the disease [14]. Older family caregivers might have limited chances to acquire appropriate knowledge about dementia due to low computer literacy. Family caregivers of people with dementia having knowledge about the disease is associated with positive attitudes toward dementia [15], and caregivers with this knowledge are more likely to engage in health promotion behaviors [16].

The fear of dementia in elderly individuals is greater than their fear of cancer [17]. Caring for family members with dementia might increase a fear of dementia, especially among older adults, because the risk of developing dementia increases with age. When elderly individuals care for people with dementia, they are more likely to have increased anxiety about developing dementia [18]. In addition, the risk of Alzheimer’s disease, one type of dementia, is associated with family history [19], which might increase the fear of dementia among family caregivers. With increasing interest in family caregivers of people with dementia, many studies have been conducted. However, most studies have focused on accumulating knowledge on their burden, psychological health issues such as depression and anxiety [20], and coping strategies [21]. This is also true in South Korea. While there is ample evidence of the burden and psychological health issues of family caregivers of people with dementia [22], there is little information about their health seeking behaviors. Thus, in this study, we explored factors that influenced the health promotion behaviors of older family caregivers of people with dementia.

## 2. Materials and Methods

### 2.1. Design, Sample, and Setting

A cross-sectional study design was adopted to identify factors that influenced health promotion behavior among older family caregivers of people with dementia. A convenient sampling technique was used at a neurology department outpatient clinic of a university hospital in the capital city of South Korea to recruit (a) older adults who were over 60 years old, and (b) who had the main responsibility of caring for family members with dementia who were living in the community. G-power 3.1 [23] was used to calculate the sample size. Using 13 independent variables with a significance level set at 0.05, a power of 0.8, and an effect size of 0.15, the appropriate sample size was 131. A total of 164 participants were recruited. After excluding 29 participants who had missing data, survey data from 135 participants were used for data analysis (response rate, 79.9%).

### 2.2. Procedure and Ethical Consideration

This study was approved by the institutional review board of the investigator’s institute. One of the researchers approached the participants to explain the study. Informed written consent was obtained from the participants before conducting the survey. Participants took 10–15 min to fill out the survey.

### 2.3. Measurements

1. Sociodemographic and caregiver-related characteristics. Items for sociodemographic characteristics included age, sex, marital status, educational status, monthly income, comorbidity condition, relationship with the patient, duration of caregiving, average time of caregiving per day, cohabitation with the patient, and usage of day care services. Comorbidities were measured with the Charlson Comorbidity Index [24], which estimates the severity of comorbidities with 17 classes of diseases weighted differently. The possible scores range from 0–32 where the higher the score, the more severe the comorbidities.
2. Dementia knowledge. Knowledge of dementia was measured with a 15-item questionnaire [25] that inquired about the causes (5-item), prevalence and policy (3-item), symptoms and diagnosis (4-item), and treatment and prevention (3-item) of dementia. All items were dichotomous with yes or no answers. Correct answers were scored as one point, and incorrect answers were scored as zero points. Possible scores range from 0 to 15 with higher scores indicating higher knowledge for dementia.
3. Fear of dementia. This concept was measured by a 30-itme questionnaire with a 5-point Likert type rating originally developed by French et al. [26], and revised and translated into Korean [27]. The possible scores range from 0 to 120, with higher scores indicating increased fear of dementia. Cronbach’s α was 0.97 in this study.
4. Health promotion behavior. Health promotion behavior was measured with the Health Promotion Lifestyle Profile-II developed by Walker [28] and translated by Seo [29]. This survey consists of 50 items with 4-point Likert type ratings. Subscales for this measure include health responsibility (8-items), physical activity (8-items), nutrition (9-items), spiritual growth (9-items), interpersonal relations (8-item), and stress management (8-items). The possible scores range between 50 and 200, with higher scores indicating greater health promotion activity. Cronbach’s α was 0.96 in this study.

### 2.4. Statistical Analyses

SPSS 26 (IBM SPSS Inc, Chicago, IL, USA) was used to perform the data analysis. Descriptive statistics were used to describe the participants’ sociodemographic and caregiver-related characteristics and variables. For the univariate analysis, the two-sample *t*-test and ANOVA were used to identify the differences in health promotion behaviors according to the sociodemographic and caregiver-related characteristics. The Scheffé test was used as a post-hoc analysis. Pearson’s correlation coefficient analysis was used to examine the relationships among study variables. Finally, hierarchical multiple regression analysis was used to identify factors that predicted health promotion behavior among participants.

## 3. Results

### 3.1. Study Variables

A description of study variables is provided in Table 1.

### 3.2. Differences in Health Promotion Behaviors

Differences in health promotion behavior according to the sociodemographic and caregiver-related characteristics are described in Table 2. Health promotion behaviors differed significantly according to age (F = 4.93, *p* = 0.009), sex (t = 2.41, *p* = 0.018), educational level (F = 4.11, *p* = 0.019), monthly income (F = 3.16, *p* = 0.027), relationships with family members with dementia (F = 6.30, *p* = 0.001), and cohabitation with family members with dementia (t = −2.13, *p* = 0.035). The *t*-test revealed that participants who were women and who were not living with family members with dementia scored higher in health promotion behavior than their counterparts. Using the Scheffé test, further differences were identified for the ANOVA results. Participants who were in their 60s had higher health promotion behavior scores than those who were older than 80 years. Participants who were high school and college graduates had higher scores in health promotion behavior than those who were elementary or middle school graduates. Participants who had a monthly income between 2000 and 3000 dollars had higher scores in health promotion behavior than those who had a monthly income less than 1000 dollars. Participants who were daughters-in-law had higher scores in health promotion behavior than participants who were spouses.

### 3.3. Correlations among Study Variables

Correlations among study variables are presented in Table 3. Health promotion behavior was positively correlated with dementia knowledge (r = 0.357, *p* < 0.001), while it was negatively correlated with fear of dementia (r = −0.546, *p* < 0.001).

### 3.4. Factors Influencing Health Promotion Behavior

For the multivariate analysis, hierarchical multiple regression analysis was conducted to identify factors that influenced health promotion behavior (Table 4). For model I, sociodemographic variables and caregiver-related characteristics were entered. Model I was not statistically significant (F = 1.46, *p* = 0.112). Model II, which included the study variables as well as those in Model I, 33.9% of the variance in health promotion behavior was explained (F = 4.271, *p* < 0.001). Sex (β = 0.210, *p* = 0.025), duration of caregiving (β = 0.197, *p* = 0.015), use of long-term care service (β = 0.180, *p* = 0.037), and fear of dementia (β = −0.558, *p* < 0.001) predicted health promotion behavior among older adults caring for family members with dementia.

## 4. Discussion

In this study, factors that influenced health promotion behavior among older adults caring for family members with dementia were identified. In our univariate analysis, those who were living with family members with dementia had a significantly lower level of health promotion behavior than their counterparts. However, the level of health promotion behavior did not differ significantly by the duration of caregiving, time spent caregiving, or usage of community support services. From this result, we can infer that living with family members with dementia rather than the intensity of caregiving time and effort influences older adults’ level of engagement in health promotion. This finding could be further elucidated with a qualitative study about living with and caring for partners with dementia. Spouses of people with dementia reported that their daily lives gradually changed based on the disease progression of dementia [30]. Furthermore, in another study, family caregivers disclosed that they voluntarily changed their lifestyles based on the needs of their family members with dementia [31]. As individual lifestyles change based on their family members’ needs when they are living with family members with dementia, health promotion of family caregivers might turn into a low priority. A family-centered tailored care plan is needed to aid family caregivers in health promotion.

When the correlations among study variables were assessed, health promotion behaviors were negatively correlated with a fear of dementia. Fear of dementia might lead to avoidance and increased anxiety [32], which could lead to negative influences on health promotion. Health promotion behaviors were positively correlated with dementia knowledge. This finding is consistent with previous studies that knowledge of dementia is associated with health promoting behavior among caregivers [16].

Our final multiple regression model revealed that sex, duration of caregiving, use of long-term care service, and fear of dementia predicted health promotion behavior. It is expected that female sex is a predictor for health promotion behaviors because sex has been consistently reported as a predictor of health promotion behaviors [33]. Usage of a senior care center positively influenced the engagement of older adults in health promoting behaviors in the model. Consistent with this finding, family care givers of patients with dementia who utilized community support services had a greater level of health promotion behaviors for physical activity, nutrition, and sleeping compared to those who utilized home care services [34]. Barriers to health promotion could be assessed to develop a tailored program for older family caregivers of people with dementia who utilized in-home services compared with those who utilized out-of-home services. Interestingly, although the impact was small, duration of caregiving had a positive influence on health promotion behavior. This might be because as time goes by, family caregivers become used to the daily routines of caring for family members with dementia and can afford health promotion for themselves. However, further studies need to be performed to explore this phenomenon.

The most influential factor for health promotion behavior among older adults caring for family members with dementia was fear of dementia when other variables were considered. Older adults who had increased fear of dementia were less likely to engage in health promotion behavior. This finding supports the findings of a previous study that showed that decreased anxiety about developing dementia was associated with an increased likelihood of engaging in health promoting behavior among elderly individuals [35]. Fear of dementia among family caregivers of patients with dementia was also negatively associated with social comfort [36], which acts as a barrier to seeking health promotion. Family caregivers are known to have increased fear of dementia, and tailored programs to alleviate the fear of dementia should be developed for older adults caring for family members with dementia.

We would like to point out that in both the univariate and multivariate analyses, the participants’ comorbidity scores did not have a significant influence on health promotion behavior. In contrast to this finding, older adults have reported that having a health problem was a barrier to physical activity [37,38,39]. The fact that 80.7% of our sample scored 0 from the comorbidity index should be considered when interpreting this finding. In addition, this might be that because the daily lives of older adults caring for family members with dementia are shaped by the patients’ status, but not by the caregivers themselves; thus, their health conditions might have a minimal influence on health promotion.

Although the results of this study are meaningful in that a strong association of a cognitive aspect, fear of dementia, was identified as a barrier to health promotion among older adults caring for family members with dementia, the following limitations should be considered. Information regarding the status of the participants’ family members with dementia was limited. The disease progression of patents [40] might contribute to the health promotion behaviors of family caregivers.

## 5. Conclusions

In this study, factors that influenced health promotion behaviors among older adults caring for family members with dementia were identified. Our univariate analysis showed that health promotion behaviors differed by age, sex, educational level, monthly income, relationship with the family member with dementia, and cohabitation with family members with dementia. Our multivariate analysis showed that the most influential factor that predicted health promotion behavior was fear of dementia. Sex, duration of caregiving, and use of long-term care service also predicted health promotion behavior. A tailored program to alleviate fear of dementia should be developed for a targeted population considering sex, the duration of caregiving and use of community support services. Cognitive behavior therapy and an online program could be used for caregivers to share their feelings about fear of dementia, followed by interactive sessions with health professionals to provide information on dementia. Thus, tailored health promotion programs for older adults might have the potential to increase health promotion.

## Figures and Tables

**Table 1:** Study variables (*N* = 135).

**Variable**	**Categories**	***n*** **(%)**	**Mean ± SD**	**Possible Range of Scores**
Age (years)	60–69	95 (70.4)	68.17 ± 7.24	
	70–79	23 (17.0)		
	≥80	17 (12.6)		
Sex	Female	66 (48.9)		
	Male	69 (51.1)		
Marital status	Married	115 (85.2)		
	Single	9 (6.6)		
	Divorced/widowed	11 (8.2)		
Highest level of education	Elementary or middle school	21 (15.6)		
	High school	49 (36.3)		
	College	65 (48.1)		
Monthly income (USD)	≤1000	35 (25.9)		
	1000–2000	28 (20.7)		
	2000–3000	33 (24.5)		
	>3000	39 (28.9)		
Comorbidity index	0	109 (80.7)		
	1	19 (14.1)		
	2	4 (3.0)		
	3	3 (2.2)		
	4–32	0 (0)		
Relationship with people with dementia	Adult children	76 (56.3)		
	Spouse	39 (28.9)		
	Daughter-in-law	16 (11.9)		
	Son-in-law	4 (3.0)		
Duration of caregiving (months)	<12	10 (7.4)	62.25 ± 53.90	
	12–24	12 (8.9)		
	24–36	18 (13.3)		
	36–60	42 (31.1)		
	60–120	26 (19.3)		
	≥120	27 (20.0)		
Time spent for caregiving (hours/day)	≤2	23 (17.0)	12.61 ± 9.19	
	2–4	14 (10.4)		
	4–8	23 (17.0)		
	8–12	17 (12.6)		
	>12	58 (43.0)		
Living with people with dementia	Yes	81 (60.0)		
	No	54 (40.0)		
Use of community support services	Adult daycare center	25 (18.5)		
	Home care service	22 (16.3)		
	Senior care center	7 (5.2)		
	None	81 (60.0)		
Dementia knowledge			10.53 ± 1.69	0–15
Fear of dementia			44.77 ± 28.35	0–120
Health promotion behavior			120.13 ± 28.46	50–200

**Table 2:** Differences in health promotion behavior according to the sociodemographic and caregiver related characteristic (*N* = 135).

**Variables**	**Categories**	**Health Promotion Behavior**			
		**Mean (SD)**	**t/F**	***p***	**Scheffé Test**
Age (years)	60–69 (a)	123.86 (26.51)	4.933	0.009 **	a > c
	70–79 (b)	118.78 (30.89)			
	≥80 (c)	101.06 (29.47)			
Sex	Female	126.05 (27.93)	2.405	0.018 *	□
	Male	114.46 (27.99)			
Marital status	Married	119.60 (28.33)	0.25	0.776	□
	Single	126.67 (31.23)			
	Divorced/widowed	120.27 (29.62)			
Highest level of education	Elementary or middle school (a)	105.29 (30.50)	4.108	0.019 *	c > a
	High school (b)	119.65 (26.61)			
	College (c)	125.28 (27.83)			
Monthly income (USD)	≤1000 (a)	110.97 (30.82)	3.162	0.027 *	c > a
	1000–2000 (b)	114.50 (31.93)			
	2000–3000 (c)	129.12 (22.90)			
	>3000 (d)	124.77 (25.43)			
Comorbidity index	0	121.93 (28.79)	1.095	0.354	□
	1	109.20 (28.28)			
	2	123.75 (20.25)			
	3	118.60 (19.21)			
	4–32	0 (0)			
Relationship with people with dementia	Adult children (a)	123.89 (25.93)	6.301	0.001 **	c > b
	Spouse (b)	105.67 (30.68)			
	Daughter-in-law (c)	136.75 (23.33)			
	Son-in-law (d)	123.00 (12.32)			
Duration of caregiving (months)	<12	126.90 (21.36)	0.427	0.829	□
	12–24	128.33 (32.67)			
	24–36	116.78 (21.58)			
	36–60	120.43 (28.34)			
	60–120	118.62 (29.07)			
	≥120	117.19 (33.36)			
Time spent for caregiving (hours/day)	≤2	132.61 (21.66)	1.564	0.188	□
	2–4	119.00 (21.79)			
	4–8	121.35 (31.09)			
	8–12	118.76 (29.72)			
	>12	115.36 (30.10)			
Living with demented family member	Yes	115.93 (28.78)	−2.127	0.035 *	□
	No	126.43 (27.01)			
Use of community support services	Adult day care center	117.92 (25.21)	0.688	0.561	□
	Home care service	125.36 (30.19)			
	Senior care center	130.43 (20.32)			
	None	118.49 (29.57)			

* *p* < 0.05, ** *p* < 0.01.

**Table 3:** Correlations among study variables (*N* = 135).

**Variable**	**Dementia Knowledge**	**Fear of Dementia**	**Health Promotion Behavior**
Dementia knowledge	1		
Fear of dementia	−0.374 **	1	
Health promotion behavior	0.357 **	−0.546 **	1

** *p* < 0.01.

**Table 4:** Hierarchical multiple regression (*N* = 135).

**Blocks of Predictors**	**Model I**				**Model II**			
	**B**	***β***	**t**	*****p*****	**B**	***β***	**t**	***p***
First block								
Age	0.18	0.05	0.31	0.755	0.56	0.14	1.12	0.264
Sex								
Female	18.85	0.33	3.12	0.002 *	11.94	0.21	2.27	0.025 *
Marital status								
Single	12.32	0.11	1.14	0.257	2.00	0.02	0.22	0.828
Divorced/widowed	5.13	0.05	0.55	0.586	2.33	0.02	0.29	0.771
Highest level of education								
High school	9.30	0.16	1.14	0.258	10.19	0.17	1.47	0.144
College	9.94	0.17	1.07	0.285	7.91	0.14	1.00	0.318
Monthly income (Dollar)								
1000–2000	2.38	0.03	0.31	0.754	4.77	0.07	0.73	0.466
2000–3000	13.03	0.20	1.65	0.102	6.87	0.10	1.03	0.307
>3000	11.90	0.19	1.25	0.214	7.05	0.11	0.88	0.382
Comorbidity index	−2.35	−0.05	−0.57	0.568	0.50	0.01	0.14	0.885
Relationship with people with dementia								
Adult children	3.88	0.07	0.42	0.676	3.47	0.06	0.45	0.656
Daughter-in-law	7.52	0.08	0.64	0.521	6.83	0.07	0.70	0.488
Son-in-law	9.95	0.06	0.59	0.555	−1.85	−0.01	−0.13	0.897
Duration of caregiving(months)	0.04	0.08	0.86	0.389	0.10	0.20	2.46	0.015 *
Time spent for caregiving (hours/day)	−0.43	−0.14	−0.89	0.373	0.16	0.05	0.38	0.703
Living with people with dementia								
No	−2.67	−0.05	−0.30	0.767	9.66	0.17	1.25	0.215
Use of community support services								
Home care service	4.85	0.06	0.55	0.582	−5.97	−0.08	−0.79	0.430
Senior care center	21.65	0.17	1.64	0.104	23.61	0.18	2.11	0.037 *
None	6.73	0.12	0.95	0.342	−2.36	−0.04	−0.39	0.697
Second block								
Dementia knowledge					1.90	0.11	1.37	0.175
Fear of dementia					−0.56	−0.56	−6.08	<0.001 **
F (*p*)	1.46 (0.112)				4.27 (<0.001 *)			

* *p* < 0.05, ** *p* < 0.01.

## Data Availability

The datasets analyzed during the current study are not yet publicly available but are available from the corresponding author on reasonable request.
